# The Effects of Tumstatin on Vascularity, Airway Inflammation and Lung Function in an Experimental Sheep Model of Chronic Asthma

**DOI:** 10.1038/srep26309

**Published:** 2016-05-20

**Authors:** Joanne Van der Velden, Louise M. Harkness, Donna M. Barker, Garry J. Barcham, Cathryn L. Ugalde, Emmanuel Koumoundouros, Heidi Bao, Louise A. Organ, Ana Tokanovic, Janette K. Burgess, Kenneth J. Snibson

**Affiliations:** 1 Faculty of Veterinary and Agricultural Science, University of Melbourne, Parkville, Australia; 2 Woolcock Institute of Medical Research, University of Sydney, NSW, Australia; 3 Discipline of Pharmacology, The University of Sydney, NSW, Australia; 4 Department of Electrical and Electronic Engineering, University of Melbourne, Parkville, Australia; 5 University of Groningen, University Medical Center Groningen, Department of Pathology and Medical Biology, Groningen, The Netherlands

## Abstract

Tumstatin, a protein fragment of the alpha-3 chain of Collagen IV, is known to be significantly reduced in the airways of asthmatics. Further, there is evidence that suggests a link between the relatively low level of tumstatin and the induction of angiogenesis and inflammation in allergic airway disease. Here, we show that the intra-segmental administration of tumstatin can impede the development of vascular remodelling and allergic inflammatory responses that are induced in a segmental challenge model of experimental asthma in sheep. In particular, the administration of tumstatin to lung segments chronically exposed to house dust mite (HDM) resulted in a significant reduction of airway small blood vessels in the diameter range 10^+^–20 μm compared to controls. In tumstatin treated lung segments after HDM challenge, the number of eosinophils was significantly reduced in parenchymal and airway wall tissues, as well as in the bronchoalveolar lavage fluid. The expression of VEGF in airway smooth muscle was also significantly reduced in tumstatin-treated segments compared to control saline-treated segments. Allergic lung function responses were not attenuated by tumstatin administration in this model. The data are consistent with the concept that tumstatin can act to suppress vascular remodelling and inflammation in allergic airway disease.

Angiogenesis is a prominent feature of airway remodelling in asthma. In particular, the airways of people with asthma have an increased number and size of blood vessels compared to healthy subjects^1^,^2^,^3^. The increased number and/or dimensions of pulmonary vessels in the bronchial mucosa can contribute to lumen narrowing and airflow obstruction in asthma^4^, and may also result in an increase in the trafficking of inflammatory cells and mediators to affected airways^5^. The precise mechanisms that lead to vascular remodelling in asthma are not known, although ongoing airway inflammation is thought to be a contributing factor^6^.

Vascular remodelling is a vital part of normal wound repair and as such is a tightly regulated process of vessel growth and regression. This process is directed by a balance between endogenous pro- and anti-angiogenic factors. Pathological angiogenesis is thought to occur when this balance is disrupted. Angiogenic factors such as vascular endothelial growth factor (VEGF), angiogenin and monocyte chemotactic protein-1 are increased in the bronchoalveolar lavage (BAL) fluid and induced sputum of asthmatics^7^. In addition to the increased levels of pro-angiogenic factors, the endogenous anti-angiogenic factors are also thought to be altered in the airways of asthmatic patients^8^,^9^. Tumstatin, the non-collagenous (NC)1 domain that can be cleaved from the α-3 chain of Collagen IV, is one such endogenous angiogenic inhibitor. We have recently reported that tumstatin is significantly reduced in the airways of patients with asthma^9^. Furthermore, administration of tumstatin in a murine model of ovalbumin-induced allergic airways disease inhibited angiogenesis, reduced airway responsiveness and dampened inflammation, suggesting that tumstatin may be a useful therapeutic intervention in asthma and other diseases where aberrant angiogenesis is a prominent feature^9^.

We have previously shown that chronic challenges with House Dust Mite (HDM) allergen to sheep lung segments induces Th-2 driven allergic inflammation which is associated with long-term lung function deterioration and small airway wall remodelling^10^,^11^. The small airway remodelling changes in the sheep include: augmented sub-epithelial collagen deposition, increases in airway smooth muscle (ASM) content, and increases in airway vascularity and blood vessel density^11^,^12^. Our recent study shows that the vascular remodelling changes in response to repeated allergen exposure are long-lasting, whereby blood vessel density in the airway walls does not revert back to control levels after allergen exposure is ceased for an extended period of time^12^. The changes to the vasculature and ASM in response to allergen, together with the fact that small airway structure and function in the sheep lung is comparable to the human lung, make the model relevant for examining the effects of tumstatin administration on vascular remodelling in asthma. Hence, the aim of the present study was to investigate whether the efficacy of tumstatin at impeding vascular remodelling and reducing airway inflammation reported for the mouse model could be verified in a large animal model of allergic asthma.

## Materials and Methods

### Experimental animals and allergen sensitisation

Female merino-cross ewes (6 months) were immunised subcutaneously with HDM extract (*Dermatophagoides pteronyssins*; CSL) and atopic sheep selected as described previously^13^. All experimental animal procedures and the collection of tissues/cells were carried out in accordance with the relevant guidelines and regulations required by the Animal Experimentation Ethics Committee of the University of Melbourne which approved this study.

### Treatment

**Figure 1:**
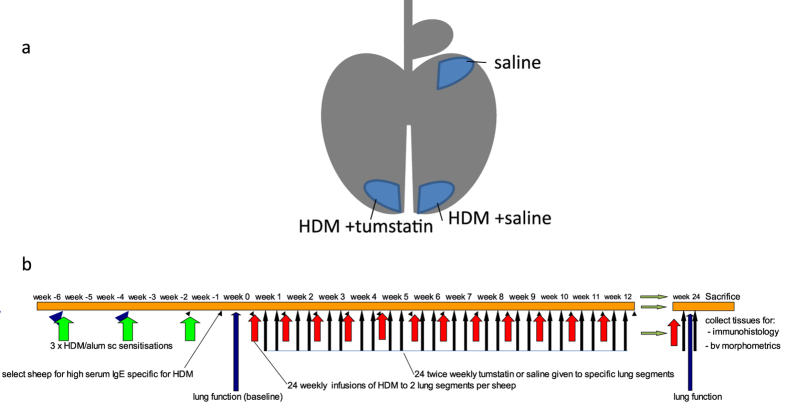
Lung segments used and timeline for procedures and measurements performed on the sheep. (**a**) A sheep lung which has been labelled to show the different lung segments used for the different treatments. The left and right distal lung segments received challenges of solubilized house dust mite (HDM) (200 μg in 5 mL saline) weekly and treatment with either Tumstatin (150 μg in 5 mL saline) or saline (5 mL) twice weekly respectively. The right medial segment received an equivalent volume of saline to act as a control for HDM. (**b**) Shows the time-line for all treatments and procedures performed on the sheep.

A fibre-optic bronchoscope was used for the localised delivery of solutions to individual lung segments. Three spatially separate lung segments received different treatments as outlined in Fig. 1. The left and right caudal lung segments received weekly challenge with HDM (1 mg in 5 mL saline) and twice weekly infusions of either tumstatin (150 mg in 5 mL saline) or saline (5 mL) for 24 weeks. The right medial segments received weekly challenges of 5 mL saline to act as a control for HDM.

### Bronchoalveolar lavage

Bronchoalveolar lavage (BAL) was collected from individual lung segments via slow infusion and withdrawal of 10 mL of sterile saline through the working channel of the bronchoscope. The samples were collected in week 22 before, and 48 hours after HDM or saline challenges to the various lung segments (Fig. 1). BAL fluid was collected and stored at −80 ^o^C until required. Total and differential cell counts were determined as previously described^14^.

### Lung function

Peripheral resistance (R_p_) of individual lung segments was measured in individual lung segments of awake, consciously breathing sheep using a wedged-bronchoscope technique and custom-built Airway Monitoring System as previously described^14^. R_p_ was calculated as mean pressure/flow during tidal breathing when pressure had stabilised. Early-phase airway response (EAR) following HDM challenge and airway responsiveness to methacholine were assessed as previously described^14^.

### Lung tissue collection, immunohistochemistry and morphometric analysis

Sheep were euthanised 7 days after the final challenge by intravenous barbiturate overdose. Lung segments from all treatment groups were inflated with optimal cutting temperature compound (Pro Sci Tech) diluted in saline (1:1). Blood vessels were identified on frozen sections of lung tissue immunostained with an antibody against type IV collagen (Dako) as previously described^12^. The density and size of blood vessels within the lamina propria of the airway wall was determined by digital image analysis of photographs using Image-Pro Plus (Media Cybernetics, version 4.1.0.0). The area of the lamina propria was defined as the area enclosed by the basal surface of the luminal epithelial cells and the luminal side of the smooth muscle bundles, using previously described nomenclature^15^. Two bronchi from the same position in the tracheobronchial tree, approximately 1–2 mm diameter and free from branching, were analysed for each segment and the results averaged for each sheep. For comparative analysis of blood vessel size, blood vessels were sorted into four different size groups based on their diameters. The cohorts of blood vessel diameter ranges were 0–10 μm, 10^+^–20 μm, 20^+^–30 μm and 30^+^ μm. Immuno-stained leukocytes were counted in the airway lamina propria and lung parenchyma at x400 magnification using a calibrated grid of known area in the microscope eyepiece. All morphometric analyses were performed by observers who were blinded to the experimental group and lung segment.

### Immunohistochemistry

Immunohistochemistry was performed on frozen tissue sections as previously described^12^,^16^. Sections were fixed with 100% cold ethanol for 10 minutes and were simultaneously blocked for endogenous peroxidase with 3% H_2_O_2_ (Univar, Knoxville, Vic, Australia). Sections were then pre-blocked using blocking solution for 30 minutes (1% bovine serum albumin, 5% normal sheep serum in PBS). After washing, sections were incubated at RT for 2 hours with the primary antibodies (e.g. anti- VEGF (A-20) rabbit polyclonal, 1:200; Santa Cruz Biotechnology; mouse anti-human type IV collagen (1:100 Dako, Kingsgrove, Australia)) and leukocyte monoclonal antibody markers for CD4, CD8, gamma delta T cell receptor and eosinophils^13^. After washing, sections were incubated for with appropriate secondary antibodies (goat anti-rabbit HRP-conjugated secondary antibody, 1:100 Dako; rabbit anti-mouse HRP-conjugated secondary antibody 1:100 Dako) for 1 hour. Sections were then washed and a peroxidase-based detection system was used for visualisation. Antibody specificity was determined by omission of the primary antibodies.

### Quantification of VEGF staining

Expression levels of VEGF staining within the airway smooth muscle were quantified calculating the intensity of the brown staining within these regions using Fuji (Image J)^17^. Airway smooth muscle bundles were outlined within complete airways and images used for analysis underwent colour deconvolution to separate the brown staining from the counterstain. Thresholds for colour (pixel) intensity were applied to images to detect the intensity of brown staining (0–166 pixels) and a threshold of 0–255 (total pixels) was applied to measure the total area of the region. For each airway, ASM bundles were outlined and area measured (Threshold 255). The outlined ASM bundles were then measured for VEGF staining (Threshold 160). To calculate Mean intensity of VEGF staining in ASM bundles, values at 160 threshold were subtracted from total area (threshold 255) i.e. ‘actual mean intensity of ASM (255–160)’.

### Statistical analyses

Differences between the segments were assessed using repeated measures (matched) one way ANOVA and the Bonferroni test for planned comparisons. Differences between 0 and 48 hour BAL cell counts were analysed for each segment using a paired (matched) t-test. Statistical analysis was performed using GraphPad Prism for Windows (Version 6.0 GraphPad Software Inc., La Jolla, USA). A P value of <0.05 was taken as significant. Values are reported as mean ± SEM.

## Results

### The Early Asthmatic Response (EAR)

**Figure 2:**
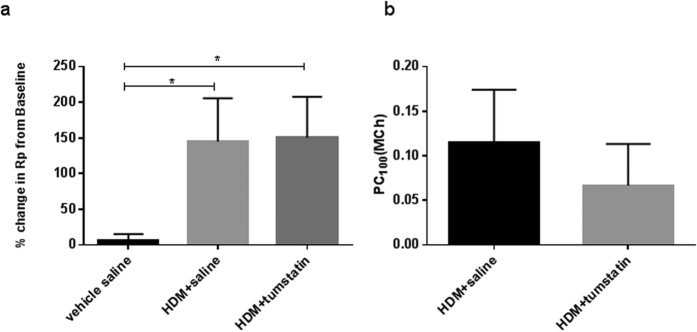
Airway responsiveness to allergic and non-allergic stimuli at week 24. **(a)** Percent change in airway peripheral airway resistance (R_p_) 30 mins post-HDM from the resistance after saline. *p < 0.05. (**b**) Percent dose of methacholine required to increase R_p_ 100% from the resistance after saline (PC_100_) 24 hours following HDM challenge. Mean + SEM (n = 7 sheep).

The Early Asthmatic Response (EAR) data shows that administering HDM alone (without tumstatin) resulted in a 148% increase in resistance in the treated segment at the 30 minute post-allergen challenge time-point when compared to baseline pre-allergen challenge resistance values (Fig. 2a). With tumstatin treatment, the mean change in segmental resistance after HDM allergen exposure was 152% above baseline values for the 30 minute time-point (Fig. 2a). There were no significant differences in the resistance responses between tumstatin- and saline control- treated segments after allergen administration to these lung compartments (Fig. 2a). As expected, control lung segments receiving saline challenges alone (without HDM) did not exhibit any significant increases in segmental resistance at the 30 minute time-point post saline challenge (Fig. 2a). Mean baseline resistances in all differentially treated lung segments at week 0 (before the start of the repeated weekly HDM challenge regime) were not significantly different to the respective baseline resistance data assessed at week 23 (Table S1, supplementary data).

### Airway responsiveness

Airway responsiveness data from week 24 of the weekly allergen challenge regime are shown in Fig. 2b. The data show PC_100_(MCh) values which indicate the relative mean dose of methacholine required to increase peripheral resistance in the nominated segment by 100% over baseline levels after control saline challenge. In general, higher PC_100_(MCh) values indicate that the airways are relatively less responsive to methacholine. The analysis demonstrated that there was no significant difference between tumstatin-treated and non-tumstatin-treated segments in terms of airway responsiveness to methacholine.

### Inflammatory cells

**Figure 3:**
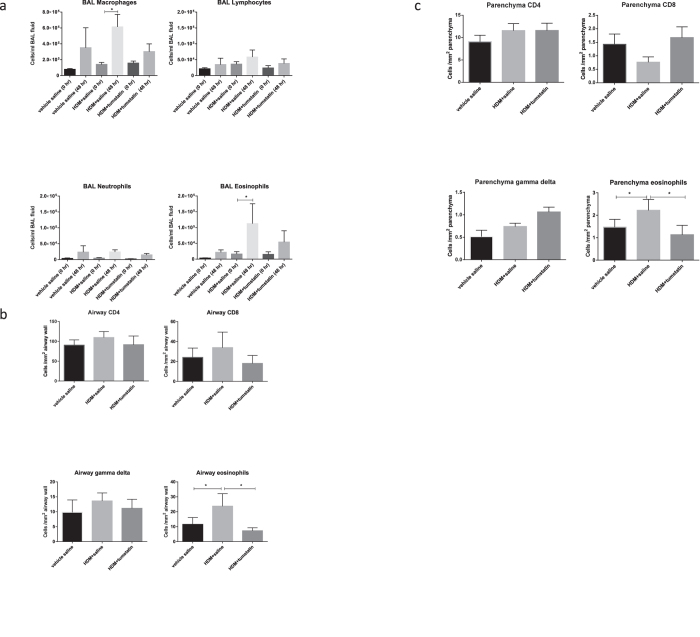
Leukocyte responses in the differentially treated lung segments. Panel (**a**) shows the numbers of leukocytes recovered from BAL fluid at week 22 of the trial both before (0 hr), and 48 hrs after, house dust mite (HDM) or saline challenges to the different segments. *p < 0.05 48 hr vs 0 hr. The data in panels (**b**,**c**) were obtained from frozen sections of the differentially treated lung segments which were immunostained for CD8- and CD4-positive lymphocytes, eosinophils, and gamma delta-positive T cell receptor cells. Panel (**b**) shows leukocyte densities in the airway wall of the differenti lung segments. Panel (**c**) shows leukocyte densities in the lung parenchyma of the different lung segments. The results are expressed as means of the differentially treatment segments. *p < 0.05 HDM + saline vs vehicle saline, and HDM + tumstatin vs HDM + saline (n = 7 sheep).

Data for leukocytes recovered from BAL fluids in week 22 is shown in Fig. 3a. The results show that the number of macrophages recovered from BAL fluid was significantly increased 48 hours after HDM +saline challenge compared with before challenge values. In contrast, with tumstatin treatment, the numbers of macrophages recovered 48 hours after HDM challenge were not significantly different from ‘before HDM challenge’ values (Fig. 3a). The numbers of eosinophils recovered from BAL fluid are significantly increased 48 hours after HDM +saline challenge compared with before challenge values. In the segment treated with tumstatin, the numbers of eosinophils recovered 48 hours after HDM challenge was higher than, but not significantly different from, ‘before HDM challenge’ values (Fig. 3a). Data for leukocyte densities (CD8, CD4, gamma delta T cell receptor, and eosinophils) in the airway walls of the three differentially treated lung segments are shown in Fig. 3b. These segments were collected at autopsy 7 days after the last HDM challenge. The data show that HDM administration alone induced a significant increase in the density of airway wall eosinophils compared with saline vehicle treated segments. In contrast, the density of airway wall eosinophils was significantly reduced in HDM + tumstatin treated segments compared with HDM alone treated segments (p < 0.05) (Fig. 3b). Data for the relative densities of parenchymal leukocytes in the differentially treated segments shown in Fig. 3c were consistent with the airway data. The data show that HDM administration alone induced a significant increase in the density of parenchymal eosinophils compared with saline vehicle treated segments. In contrast, the density of parenchymal eosinophils was significantly reduced in HDM + tumstatin treated segments compared with HDM alone treated segments (p < 0.05). There were no significant differences between the densities of the CD4, CD8, and gamma delta T cell receptor lymphocytes in either the airway walls, or in the parenchyma, of the differentially treated lung segments.

### Airway smooth muscle and lamina propria areas measurements

Airway smooth muscle and lamina propria areas measurements for the three differentially treated lung segments are indicated in Table S1 of the supplementary data.

### Blood vessel morphometrics

**Figure 4:**
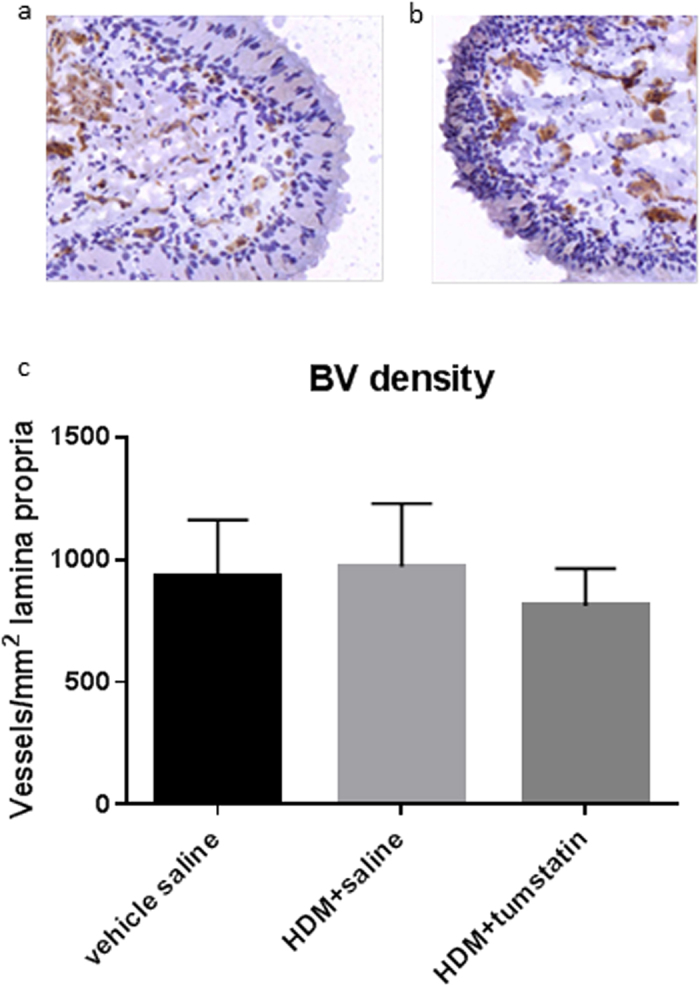
Airway blood vessel density in the differentially treated lung segments. Representative photomicrographs of blood vessels stained with Type IV Collagen are shown for (**a**) HDM + saline, and (**b**) HDM + tumstatin treated lung segments. (**c**) Shows the density of blood vessels in the airway lamina propria immuno-stained with Type IV Collagen (n = 7 sheep).

**Figure 5:**
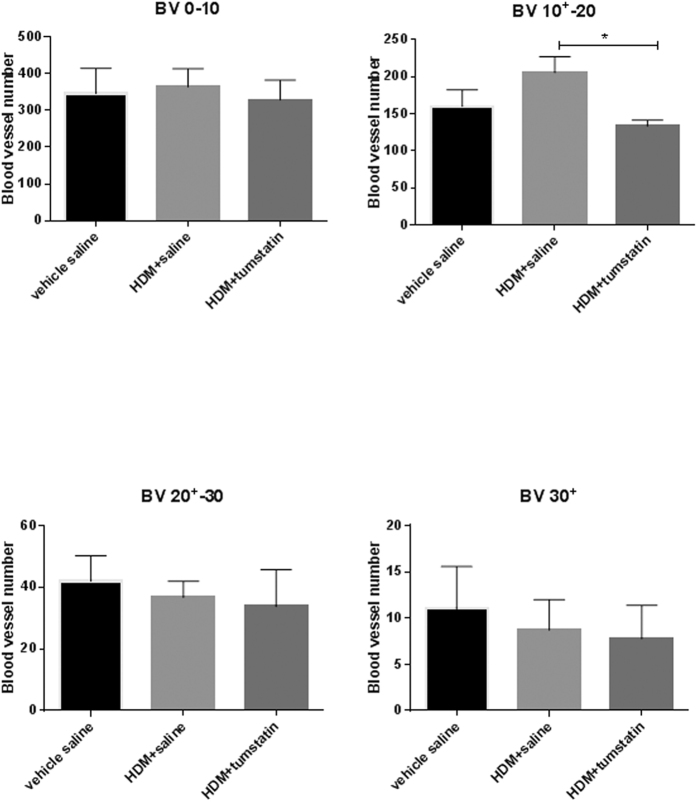
Density of different sized blood vessels in the airway lamina propria of differentially treated lung segments. Blood vessels in frozen sections were immuno-labelled with a ColIV antibody and then sorted into four different size groups based on their diameters. The diameter ranges in the groups were 0–10 μm, 10^+^–20 μm, 20^+^–30 μm and 30^+^ μm. *p < 0.05 HDM + tumsatin treatment vs HDM + saline (n = 7 sheep).

Morphometric data on bronchial blood vessels in the airway lamina propria of differentially treated lung segments are shown in Fig. 4. Representative sections of airway blood vessels stained with ColIV antibody are shown in Fig. 4a,b. The density of blood vessels of all sizes in a unit area of lamina propria (vessels/mm^2^ lamina propria) was lower, but not significantly different, in the HDM +tumstatin treated lung segments Fig. 4c. To determine whether tumstatin was having differential effects on different sizes of blood vessels represented in histological sections, we sorted blood vessels into four different size groups based on their diameters (0–10 μm, 10^+^–20 μm, 20^+^–30 μm and 30^+^ μm). A comparison between the differentially treated lung segments indicated that there was a significant difference in the density of 10^+^–20 μm size vessels between HDM+tumstatin and HDM + saline treatments (206 ± 21 v 133 ± 8 vessels/mm^2^ lamina propria respectively, p < 0.05, Fig. 5).

### VEGF expression

**Figure 6:**
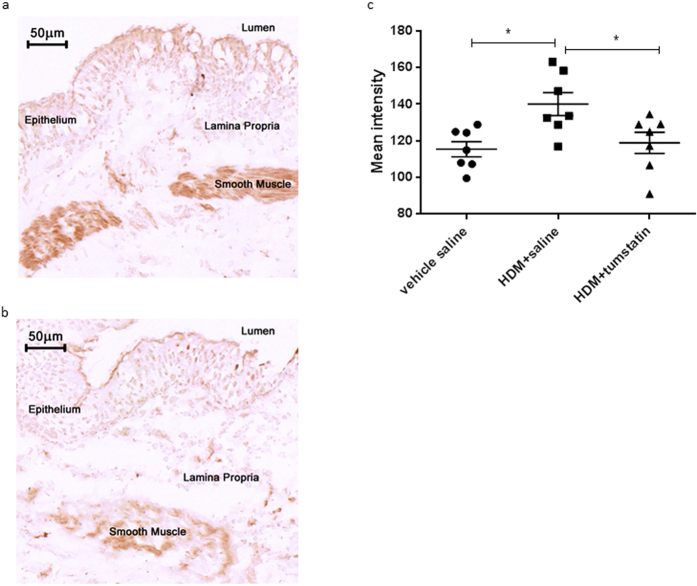
VEGF expression in ASM. Representative photomicrographs of airway sections immunostained with an antibody against VEGF for the HDM + saline treated lung segment (**a**), and the HDM + tumstatin treated lung segment (**b**). (**c**) Shows the mean intensity of VEGF immunostaining in individual sheep in the three differentially treated lung segments. *p < 0.05 (n = 7 sheep).

An analysis of VEGF immunostaining showed that VEGF was expressed predominantly in the airway epithelium and airway smooth muscle (Fig. 6a,b). Exposure to HDM, without tumstatin, significantly increased the VEGF staining within the ASM bundles compared to the vehicle control (mean intensity of VEGF 136.4 ± 7 vs 116.8 ± 5 mean intensity units respectively, p < 0.05). Treatment of the segments with HDM + tumstatin prevented the increase in VEGF staining in the ASM (mean intensity of VEGF 120 ± 11 vs 116.8 ± 5, respectively) (Fig. 6c).

## Discussion

In this study we have used our large animal model of experimental chronic asthma^12^ to explore the effects of tumstatin on the disease features that are reflective of those seen in human asthma. Our major findings were that intra-lung segmental administration of tumstatin reduced the induction of the development of new blood vessels by HDM and attenuated allergic eosinophilic and macrophage responses to HDM challenge. However, the application of tumstatin but did not alter lung function indices for either the early-phase asthmatic response or the bronchial hyperresponsiveness. These findings suggest that tumstatin may be able to prevent the *in vivo* induction of angiogenesis and the allergic inflammatory response in small airways chronically exposed to allergen.

Much of the interest in the role that tumstatin plays in asthma stems from a key study reporting that the level of tumstatin is decreased 18-fold in the airways of patients with asthma, relative to non-asthmatic subjects^9^. Small animal model data are supportive of a role for tumstatin in asthma in that tumstatin administration to mice with induced allergic airways disease suppresses angiogenesis, airway hyperresponsiveness, inflammatory cell infiltration, and mucus secretion^9^. In the current study, morphometric analyses of blood vessels indicate that tumstatin treatment of HDM challenged segments resulted in a significantly lower density of small bronchial blood vessels compared to equivalent size blood vessels in all other lung segments examined which did not receive tumstatin. The process of angiogenesis, the formation of new blood vessels from existing vessels, is a complex process that involves the activation and sprouting of the endothelial cells to form the leading tip of the new vessel. The environment around the vessel must then be remodelled before the vessel is stabilised. Endothelial cells are normally quiescent while they are bound to the capillary BM, this suggests that the initial signals coming from the BM inhibit proliferation and facilitate appropriate cell–cell adhesion (which would be seen in the very small vessels in our study). Not all new vessels mature to a stable new structure but rather the vast majority begin to form and then regress^18^. Tumstatin targets its actions through inducing apoptosis in the activated endothelial cells that are a key component during angiogenesis^19^,^20^. In this way it regulates the formation of new blood vessels, which are reflected in the 10^+^–20 μm blood vessel size cohort in the analysis in this study, but does not alter the stability of the preexisting vessels in the airways (reflected in the larger diameter groupings in our study). As such, tumstatin would usually play an important regulatory role that assists in the usual maintenance of physiological angiogenesis in the airways. The reduction in tumstatin in the airways of people with asthma indicates that this physiological balance may have been disrupted, which may explain the increases in blood vessel size and density that are present in these airways^21^,^22^,^23^.

Our results were derived using a recombinant collagen IV NCI fragment introduced into the airways of allergic sheep. One potential limitation of the study is that we cannot rule out that our data is the result of a non-specific protein fragment effect, as we did not use an inactive recombinant collagen fragment for the control lung segment. Inactive protein fragments of over 300AA are very difficult to generate for controls, and so far, to our knowledge, all collagen fragments that have been examined in *in vivo* models have been generated to investigate an active, rather than an inactive, role in the biological function. To date, studies reporting an active biological role of tumstatin have not used an inactive recombinant collagen fragment to illustrate the specificity of the response^9^,^19^,^24^. The specificity of the collagen IV NCI fragment has been shown using peptides and scrambled controls in *in vivo* models and support the hypothesis that tumstatin is the biologically active molecule^25^.

Interestingly, the analyses of lung function data in the differentially treated lung segments, show that tumstatin administration does not attenuate lung function indices of EAR and AHR in the sheep model. These results are in contrast to the data generated using the mouse allergic airways model where tumstatin improved AHR as well as a number indices of allergic inflammation^9^. This result was surprising as we expected that the tumstatin-induced changes to the vascularity and inflammation would improve airway function in the sheep model, as it does in mice. Explanations for the contrasting results may be due to factors such as differences in airway structures between the two species, as well as the very different protocols associated with measuring lung function in mice and sheep. It is noteworthy that the physiological analyses of the current study were conducted on the small airways of unanaesthetised spontaneously breathing sheep. Whereas lung function in the mouse study was conducted on whole lungs of anaesthetised mice which were mechanically ventilated^9^. The major differences in small airway structures between the species which may influence function are that small airways of sheep possess well-developed bronchial circulations, mast cells and prominent smooth muscle bundles^26^. These small airway features are either absent or poorly developed in the mouse small airway. Interestingly, in our previous publication, we found that while there were deteriorations in functional indices of EAR or AHR in lung segments of sheep chronically exposed to allergen, the changes in small airway function did not directly correlate to increased blood vessel density in response to repeated allergen challenge reported for this sheep model^12^. Thus, it could be reasonably argued that any changes to the vasculature with tumstatin administration that we see in this study are unlikely to be prominent enough to produce significant improvements in lung function. Overall, while tumstatin had beneficial effects on allergic inflammation and angiogenesis in the sheep model, the lack of improvement in pulmonary function after tumstatin administration could indicate a limited usefulness for directly improving lung function. It is worth noting that tumstatin may be effective as an add-on therapy for treating asthmatics, targeting aspect of remodeling that are not altered by the current therapeutic regime^27^,^28^.

While angiogenesis has been studied in the circulation associated with the mouse trachea^29^ and others have shown increased lung vascularity following HDM or ovalbumin exposures^30^,^31^ the relevance of these findings to human asthma is controversial due to the remodelling of airway bronchial circulation in human asthma as opposed to changes in the pulmonary circulation as seen for mice. In the sheep model, as is the case with human asthma, the blood vessels of the bronchial circulation in the airway lamina propria are associated with airway oedema and are a major entry point for inflammatory cells to migrate into other regions of the airway wall^32^,^33^. It is possible that the differences in the effectiveness of tumstatin seen in the sheep model presented here compared to data from the mouse model are reflective of the altered location of the reduction in angiogenesis, which may change the regulation of the influx of inflammatory cells into the airway wall and their impact on AHR.

We found that local tumstatin administration to specific lung segments ameliorated allergic inflammatory responses to HDM in these segments. This was evident in the lower numbers of eosinophils present in recovered BAL fluid, and lower eosinophil densities observed in parenchymal and airway wall tissues of tumstatin-treated lung segments when compared to data derived from comparable segments treated with HDM alone. In this respect, our data are consistent with earlier findings which demonstrate that intranasal administration of tumstatin in a mouse model of allergic airway disease results in reduced densities of eosinophils and airway mucous secreting cells^9^. While there is very little known with regard to its anti-inflammatory mechanism, the administration of tumstatin is known to protect the basement membrane from damage from polymorphonuclear cells^34^. Further, the addition of tumstatin inhibits the accumulation of monocytes and macrophages in a mouse model of diabetic neuropathy^24^. It is of interest that we also see reduced levels of macrophages in BAL fluid after HDM challenge and tumstatin treatment. While the above mentioned studies may suggest that tumstatin is directly anti-inflammatory, it cannot be ruled out that the amelioration of allergic inflammation is indirectly due to tumstatin’s retardation on the growth of new blood vessels which act as a conduit for entry of inflammatory cells to airway tissues.

The administration of tumstatin significantly reduced the expression of VEGF protein detected within the ASM bundles in the airways. The ASM is a known source of VEGF, releasing it in response to pro-inflammatory and pro-fibrotic factors increased in the asthmatic milieu^35^,^36^. Whilst the modulation of VEGF levels by tumstatin in the ASM has not previously been reported, to our knowledge, it is feasible that tumstatin binds to the integrins αvβ3 or αvβ5 on the ASM cell surface^37^,^38^. Through this interaction it would modulate downstream signalling in the ASM cell. It is through binding to this integrin on endothelial cells that tumstatin prevents their receiving the survival signal from VEGF and drives the endothelial cells into apoptosis^20^. These findings suggest that the effects of tumstatin in the airways go beyond the immediately recognised effects on endothelial cells. The potential for tumstatin to have broader effects on airway remodelling in the asthmatic lung require further investigation.

Overall, this large animal study confirms the important link between the absence of tumstatin and increased blood vessel growth and the inflammatory response in asthma. Our findings suggest that application of tumstatin directly to the lung may be able to prevent the *in vivo* remodelling and inflammation that is associated with chronic asthma.

## Additional Information

**How to cite this article**: Van der Velden, J. *et al*. The Effects of Tumstatin on Vascularity, Airway Inflammation and Lung Function in an Experimental Sheep Model of Chronic Asthma. *Sci. Rep.* **6**, 26309; doi: 10.1038/srep26309 (2016).

## Supplementary Material

Supplementary Information
