# Supplementary material for: The Effects of Tumstatin on Vascularity, Airway Inflammation and Lung Function in an Experimental Sheep Model of Chronic Asthma
Source: Sci Rep. 2016 May 20;6:26309. doi: 10.1038/srep26309 (PMC4873797; doi:10.1038/srep26309)
Supplement: Supplementary Information [file srep26309-s1.doc]

**The Effects of Tumstatin on Vascularity, Airway Inflammation and Lung Function in an Experimental Sheep Model of Chronic Asthma**

Joanne Van der Velden, Louise M Harkness, , Donna M Barker, Garry J Barcham, Cathryn L Ugalde, Emmanuel Koumoundouros, Heidi Bao, Louise A Organ Ana Tokanovic, Janette K Burgess, Kenneth J Snibson

| Treatment | Airway lamina propria area / unit BM length (μm^2^/μm) | Airway smooth muscle area / BM length   (μm^2^/mm) | Baseline resistance  Week 0  (cmH_2_O/l/s)  x 10^3^ | Baseline resistance Week 23  (cmH_2_O/l/s)  x 10^3^ |
| --- | --- | --- | --- | --- |
| Vehicle saline | 128 ± 18 | 59 ±6 | 21 ±3 | 25 ± 2 |
| HDM +saline | 105 ± 9 | 58 ± 7 | 5.6 ±1 | 6.7 ± 1 |
| HDM + tumstatin | 104 ± 17 | 71 ± 19 | 3.4 ± 0.7 | 7.1 ± 2 |

Supplementary Table S1 Airway morphometry and lung function analyses of the three differentially treated lung segments of 7 sheep. Airway smooth muscle and lamina propria data are expressed as area per unit length airway basement membrane (BM). Baseline lung function was assessed at week 0 before the start of the repeated weekly HDM challenge regime, and also at week 23 immediately before the 23rd weekly HDM challenge (cmH_2_O/l/s, cm H_2_O/ litre/second) Results are expressed as group means ± SEM.
